# Is catheter ablation associated with preservation of cognitive function? An analysis from the SAGE-AF observational cohort study

**DOI:** 10.3389/fneur.2023.1302020

**Published:** 2024-01-05

**Authors:** Bahadar S. Srichawla, Alexander P. Hamel, Philip Cook, Rozaleen Aleyadeh, Darleen Lessard, Edith M. Otabil, Jordy Mehawej, Jane S. Saczynski, David D. McManus, Majaz Moonis

**Affiliations:** ^1^Department of Neurology, University of Massachusetts Chan Medical School, Worcester, MA, United States; ^2^Department of Medicine, University of Massachusetts Chan Medical School, Worcester, MA, United States

**Keywords:** atrial fibrillation, cognitive function, ablation, stroke, MoCA, infarction, dementia, cognitive decline

## Abstract

**Objectives:**

To examine the associations between catheter ablation treatment (CA) vs. medical management and cognitive impairment among older adults with atrial fibrillation (AF).

**Methods:**

Ambulatory patients who had AF, were ≥65-years-old, and were eligible to receive oral anticoagulation could be enrolled into the SAGE (Systematic Assessment of Geriatric Elements)-AF study from internal medicine and cardiology clinics in Massachusetts and Georgia between 2016 and 2018. Cognitive function was assessed using the Montreal Cognitive Assessment (MoCA) tool at baseline, 1-, and 2 years. Cognitive impairment was defined as a MoCA score ≤ 23. Multivariate-adjusted logistic regression of longitudinal repeated measures was used to examine associations between treatment with CA vs. medical management and cognitive impairment.

**Results:**

887 participants were included in this analysis. On average, participants were 75.2 ± 6.7 years old, 48.6% women, and 87.4% white non-Hispanic. 193 (21.8%) participants received a CA before enrollment. Participants who had previously undergone CA were significantly less likely to be cognitively impaired during the 2-year study period (*aOR 0.70, 95% CI 0.50–0.97*) than those medically managed (i.e., rate and/or rhythm control), even after adjusting with propensity score for CA. At the 2-year follow-up a significantly greater number of individuals in the non-CA group were cognitively impaired (MoCA ≤ 23) compared to the CA-group (311 [44.8%] vs. 58 [30.1%], *p = 0.0002*).

**Conclusion:**

In this 2-year longitudinal prospective cohort study participants who underwent CA for AF before enrollment were less likely to have cognitive impairment than those who had not undergone *CA.*

## 1 Introduction

Atrial fibrillation (AF) is the most common cardiac arrhythmia in the United States, affecting 3 to 6 million people (1). The Framingham Heart Study demonstrated a 3-fold increase in the prevalence of AF in the past 50 years (2). AF carries a two-fold increased risk of mortality and a five-fold risk of ischemic stroke. AF has also been associated with ischemic phenomena and cardioembolic-mediated infarction to critical organs including the gastrointestinal tract and kidneys, among others (3). To mitigate the risk of stroke secondary to AF, anticoagulants (ACs) such as warfarin and direct oral anticoagulant (DOAC) therapy are often used based on the CHA_2_DS_2_-VASc score. The CHA_2_DS_2_-VASc score is a clinical prediction rule for estimating the risk of stroke in patients with non-rheumatic atrial fibrillation (AF), a common and irregular heart rhythm. However, the utilization of AC is not without risk. Major hemorrhagic events, including intracerebral hemorrhage (ICH), and gastrointestinal bleeding, are of concern (4).

Previous studies have shown that AF is independently associated with cognitive decline. Proposed pathophysiologic mechanisms include silent ischemic or hemorrhagic cerebral micro-infarction and/or impaired cerebral blood flow (5). Catheter ablation (CA) has become a common rhythm control management strategy for patients with AF and is the most frequently performed cardiac ablation procedure worldwide. Restoring sinus rhythm via CA addresses several of the proposed pathophysiologic mechanisms that may link AF to cognitive decline. Bodagh et al. performed a systematic review that identified 10 studies that assessed cognitive function in patients who underwent CA vs. medical management. Incident dementia was lower among patients who received CA for rhythm control of AF as compared to those treated with medicine. However, other studies involving patients with AF with a more rigorous longitudinal assessment of cognitive function have shown no relation between CA and cognitive decline (6). Finally, one of the major confounders when examining the relations between CA and AF is the use of anticoagulation, which can also have important effects on cerebrovascular health. In addition to the clear reduction in ischemic stroke attributable to anticoagulation, its use has been shown to cause cerebral microbleeds (CMBs) which may reduce cognitive function (7, 8).

In this retrospective analysis, we used data from the Systematic Assessment of Geriatric Elements in Atrial Fibrillation (or SAGE-AF) study, a prospective cohort study that enrolled individuals with AF aged ≥ 65 years and followed them over 2 years to understand the relations between oral anticoagulation, cognitive and physical function. To better understand the potential links between CA and cognitive health, we compared cognitive function at baseline and 1- and 2-year follow-up examinations among those SAGE-AF participants who underwent CA with those treated medically (either rate or rhythm control).

## 2 Methods

### 2.1 Design, setting, and participants

We examined data from the prospective cohort multicenter SAGE-AF study, whose details have been previously described (9). Briefly, the SAGE-AF study enrolled patients age ≥ 65 from ambulatory care sites in Massachusetts and Georgia from 2016 to 2018. Patients were eligible if they had a documented diagnosis of AF and a CHA_2_DS_2_-VASc (congestive heart failure, hypertension, age ≥ 75, diabetes, stroke, vascular disease, age 65–74, and sex) score ≥ 2. Exclusion criteria included being contraindicated for oral anticoagulants, being unable to provide signed informed consent in English, and being unable to participate in follow-up visits during the 2-year study period. Contraindications to anticoagulants included recent or planned major surgery, recent or active major bleeding, use of anticoagulants for reasons other than AF, or significant thrombocytopenia (platelet count < 50,000).

All participants enrolled in the study provided their informed written consent for protocols approved by the institutional review boards of the University of Massachusetts Medical School, Boston University, and Mercer University. Each participant underwent a routine physical examination, which included an electrocardiogram (ECG) at baseline and 2 years. Participants also had their medical history abstracted in addition to a 60-min computer-assisted interview that assessed mood, cognition, social support, and other key patient-reported measures using standardized measures. We specifically extracted age, sex, race, education level, cognitive ability, frailty, depression, anxiety, social isolation, and smoking status at baseline. Other factors assessed included a history of intracranial hemorrhage, GI bleeding, major bleeding, heart failure, coronary artery disease – myocardial infarction or angina, peripheral vascular disease, hypertension, type II diabetes, dyslipidemia, ischemic stroke, anemia, chronic obstructive pulmonary disease, renal disease, implantable cardiac device, and sleep apnea. Laboratory values extracted included creatinine, hemoglobin, and platelet count. The HAS-BLED score is a clinical prediction rule used to estimate the risk of major bleeding for patients who are on anticoagulation therapy, especially those with atrial fibrillation (AF). The CHA_2_DS_2_-VASc and HAS-BLED scores were calculated, and polypharmacy, utilization of anticoagulants, antiplatelet agents, and antiarrhythmics were assessed.

### 2.2 Global cognitive function assessment

Our primary outcome, cognitive function, was measured using the validated Montreal Cognitive Assessment (MoCA) tool, a 10-min and 30-item screening tool used to detect cognitive impairment (10). MoCA tests memory, visuospatial ability, executive function, attention, concentration, working memory, and orientation and is accepted for use in an older population of patients with strokes (11). Cognitive impairment was defined as a cut-off of ≤23, in accordance with prior studies (12, 13). Each participant included in this analysis completed the MoCA at baseline, 1-year, and 2-years.

### 2.3 AF treatment

As part of the medical record abstraction, study coordinators collected procedural histories of participants, including prior ablation and medication use. Participants who had previously undergone a catheter ablation prior to their baseline SAGE-AF examination were considered to have undergone CA. 29 participants were ablated before their baseline exam and received another CA during the follow-up period. The specific type of CA performed was not reported, but since all CA participants were enrolled between 2016 and 2018 from US-based clinics, the majority of CA performed during this time were likely to have been radiofrequency ablation pulmonary vein isolation procedures (14).

For each participant, medications were abstracted from the electronic medical record and confirmed with the participant during the in-person interview. Relevant medications abstracted from the health record included OAC as well as rate and rhythm control agents. Specifically, prescriptions of antiarrhythmic, rate control, antiplatelet, and anticoagulant agents (including vitamin K antagonists and DOACs) were abstracted and confirmed with participants. Participants who did not receive CA were considered as ‘medically treated’ and were further subdivided into rate or rhythm control groups, consistent with a prior SAGE-AF analysis (15). In brief, rhythm control was defined as the use of an antiarrhythmic drug or prior cardioversion, while rate control included all SAGE-AF participants who did not report treatment with an antiarrhythmic drug or cardioversion during their baseline examination.

### 2.4 Clinical outcomes

The pre-specified analysis of major adverse cardiovascular endpoints (MACE) included mortality due to cardiovascular cause (vascular death), myocardial infarction (MI), stroke, deep vein thrombosis (DVT), pulmonary embolism (PE), and major bleeding. These events were obtained from participants’ medical records, death certificates, and follow-up assessments. Major bleeding events were adjudicated by a physician committee and classified according to the International Society of Thrombosis and Hemostasis scale (16). The major bleeds were fatal, occurred in a major organ, or required more than 2 units of transfusion due to hemoglobin loss (17). A subgroup analysis was performed in both groups to assess the effects of warfarin vs. DOACs on cognitive function.

### 2.5 Quantitative variables and statistical methods

Our analytical sample of 887 included those who had completed cognitive function evaluations (described below) at baseline, 1-year, and 2-year follow-ups. Demographic and baseline clinical characteristics of the participants were compared between participants with a previous ablation procedure at the beginning of the study and participants without a history of ablation using analysis of variance for continuous variables and the Chi-square test for categorical variables. All individuals in the CA group underwent the procedure prior to enrollment in the study.

A propensity score for undergoing CA was calculated for each participant in this analysis. Variables that were included in the propensity score were sex, age, BMI, CHA_2_DS_2_-VASc score, AF type (paroxysmal, persistent, or permanent), education level, smoking status, history of hypertension, history of heart failure, history of diabetes, history of stroke, history of renal disease, history of sleep apnea, use of rhythm control drugs, use of rate control drugs, and use of oral anticoagulants. These variables were selected based on clinical relevance and significance in Table 1. Mixed-effects logistic regression was employed for longitudinal binary response data to examine the relationship between prior ablation and cognitive impairment. This relationship was adjusted for the propensity score, maintenance of normal sinus rhythm (NSR) as an indication of CA success, and treatment group (rhythm vs. rate control). The relationship between the type of anticoagulation treatment and cognitive impairment was adjusted for the propensity score only.

**Table 1:** Participant characteristics according to receipt of catheter ablation procedure for AF prior to enrollment.

Characteristics	Catheter Ablation Status at Baseline (n)		
Socio-demographics	Yes (*n* = 193)	No (*n* = 694)	*p*-value
Age, mean, years (SD)^*^	73.0 (6.1)	75.9 (6.8)	**<0.0001**
**Age, category (%)**			
65–74 years	123 (63.7)	331 (47.7)	**<0.001**
75–84 years	58 (30.1)	277 (39.1)	
85 years or older	12 (6.2)	86 (12.4)	
Female sex (%)	95 (49.2)	336 (48.4)	0.84
**Race (%)**			
Non-Hispanic White	174 (90.2)	601 (86.7)	0.20
**Education (%)**			
College graduate or more	86 (44.6)	309 (45.0)	0.90
**AF type**			
Paroxysmal	118 (61.1)	415 (59.8)	**<0.01**
Persistent	60 (31.1)	156 (22.5)	
Permanent	4 (2.1)	41 (5.9)	
Other	11 (5.7)	82 (11.8)	
**Baseline (%)**			
Cognitive impairment (MOCA ≤ 23)	62 (32.1)	274 (39.5)	0.06
Frailty			
Frail	28 (14.5)	76 (11.0)	0.36
Prefrail	94 (48.7)	364 (52.5)	
Not Frail	71 (36.8)	254 (36.6)	
Able to walk 15 ft	191 (99.0)	678 (97.7)	0.39
Depression	56 (29.0)	165 (23.8)	0.14
Anxiety	50 (25.9)	143 (20.6)	0.11
Social isolation (MOS < 12)	30 (15.5)	84 (12.1)	0.21
Smoking status			
Never smoker	95 (49.2)	332 (47.8)	0.76
Former smoker	94 (48.7)	341 (49.1)	
Current smoker	4 (2.1)	21 (3.0)	
**Bleeding history (%)**			
Intracranial hemorrhage	2 (5.6)	7 (5.7)	1.00
GI bleed	24 (64.9)	70 (56.9)	0.39
Major bleed	37 (19.2)	123 (17.7)	0.64
Heart failure	79 (40.9)	223 (32.1)	**0.02**
CAD—MI or angina	48 (24.9)	187 (27.0)	0.56
Peripheral vascular disease	20 (10.4)	104 (15.0)	0.10
Hypertension	171 (88.6)	619 (89.2)	0.81
Type II diabetes	52 (26.9)	179 (25.8)	0.75
Dyslipidemia	157 (81.4)	562 (81.0)	0.90
Ischemic stroke	13 (6.7)	71 (10.2)	0.14
Anemia	61 (31.6)	207 (29.8)	0.63
COPD	52 (26.9)	163 (23.5)	0.32
Renal disease	37 (19.2)	192 (27.7)	**0.02**
Implantable cardiac device	88 (45.6)	191 (27.5)	**<0.0001**
Sleep apnea	65 (33.7)	181 (26.1)	**0.04**
**Blood levels**			
Creatinine (mg/dL)	1.01 (0.29)	1.08 (0.49)	0.11
Hemoglobin (mg/dL)	12.9 (1.8)	13.2 (1.8)	0.06
Platelets	205.6 (63.0)	210.7 (73.2)	0.61
**Risk scores**			
CHA2DS2-VASc score (SD)*	4.24 (1.46)	4.36 (1.60)	0.50
HAS-BLED score (SD)*	3.15 (1.01)	3.21 (1.08)	0.45
**Medications (%)**			
**Total #**			
1	38 (19.7)	154 (22.2)	0.41
2	58 (30.1)	173 (24.9)	
3	41 (21.2)	173 (24.9)	
4+	56 (29.0)	194 (28.0)	
Any oral anticoagulant	173 (89.6)	585 (84.3)	0.06
Warfarin	69 (39.9)	370 (63.1)	**<0.0001**
Antiplatelet	11 (5.7)	41 (5.9)	0.91
**Treatment method**			
Rhythm control	137 (71.0)	317 (45.7)	**<0.0001**
Rate control	56 (29.0)	377 (54.3)	

Only patients with MOCA scores at all time points are included. ^*^Age value, CHA2DS-VASc Score, and HAS-BLED Score reported with no missing data. Statistically significant values bolded.

We adjusted for clinically relevant and significant Table 1 variables when examining the relationship between prior ablation and clinical outcomes. These components included age, history of CAD, implantable cardiac devices, sleep apnea, use of antiarrhythmics, and use of anticoagulants. Statistical analyses were performed using SAS c9.4 (SAS Institute Inc., Cary, NC).

## 3 Results

A total of 887 SAGE-AF participants were included in this analysis. The total number of participants at baseline are included both at the 1- and 2-year follow-up period. Participants included in our analysis were on average 75.2 ± 6.7 years old, 48.6% were women, and 87.4% self-identified as non-Hispanic white. Of the total, over half (60.1%) were classified as having paroxysmal atrial fibrillation, and 193 (21.8%) individuals had undergone a CA procedure before enrollment. 336 (37.9%) participants were cognitively impaired at baseline. The mean scores of CHA_2_DS_2_-VASc and HAS-BLED at baseline were 4.33 ± 1.57 and 3.20 ± 1.06, respectively.

Demographic and clinical characteristics by CA history are shown in Table 1. Participants who had undergone a CA procedure pre-enrollment were younger and less likely to be taking warfarin (*39.9%* vs. *63.1%, p < 0.0001*) or rate control drugs (*80.0%* vs. *80.6%, p < 0.05*) than were participants in the non-CA group. Of the CA group, 173 (89.6%) were on any OAC at baseline, compared to 585 (84.3%) of the non-CA group (*p* = 0.06). Those who had undergone a CA were more likely to have persistent AF than participants in the non-CA group (*31.1%* vs. *22.5%, p < 0.05*). Participants who were treated with CA pre-enrollment were more likely to have also undergone ICD implantation (*45.6%* vs. *27.5%, p < 0.001*), and were more likely to have comorbid heart failure (*40.9%* vs. *32.1%, p < 0.05*) and sleep apnea (*33.7%* vs. *26.1%, p < 0.05*), whereas they were less likely to have chronic renal disease (*19.2%* vs. *27.7%, p < 0.05*) when compared with those who did not receive CA (Table 1).

### 3.1 CA vs. medical management and relation to cognitive impairment

**Figure 1:**
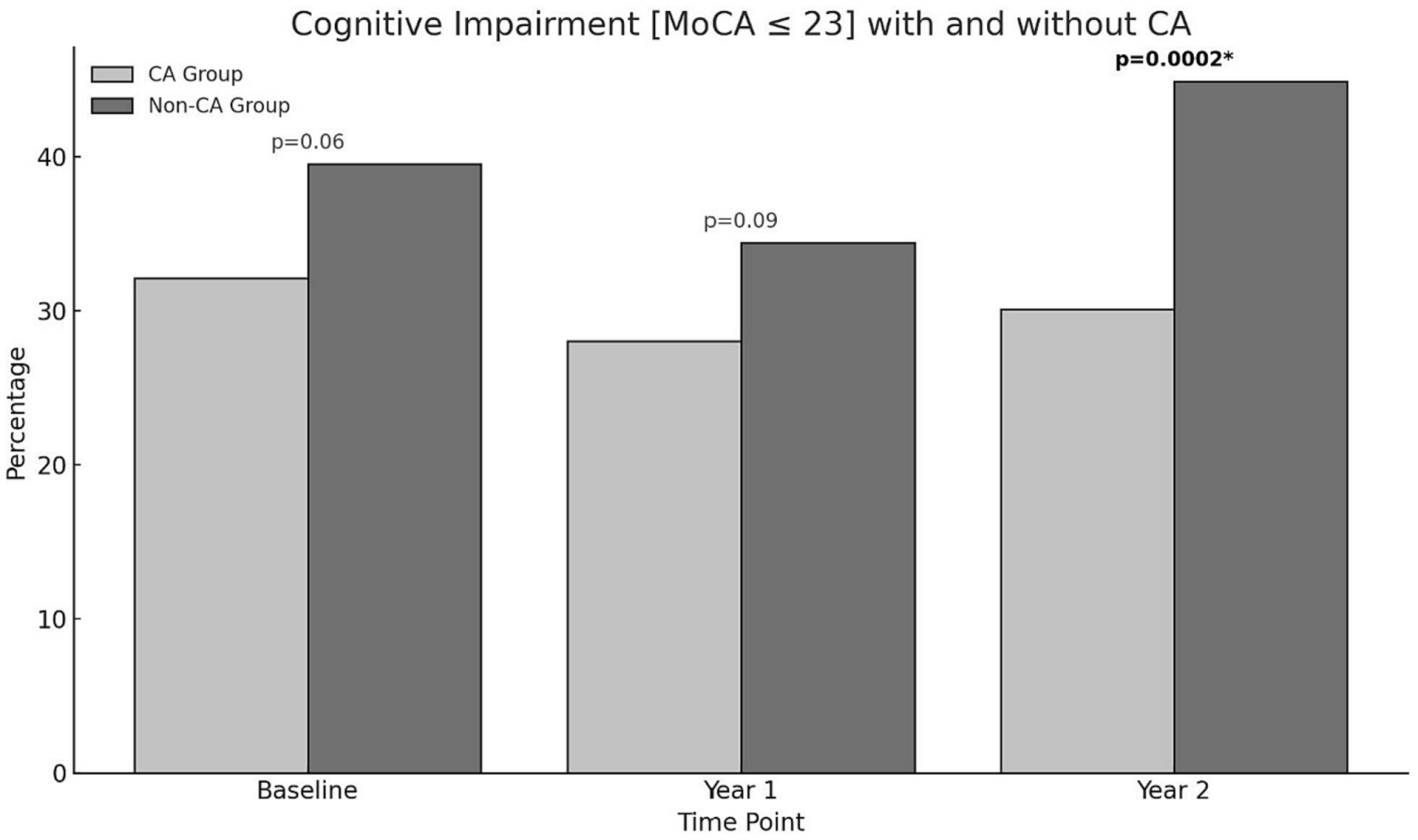
Percentage of individuals in the CA and non-CA groups with cognitive impairment defined by a MoCA ≤ 23 at baseline, 1-, and 2-year end points. At baseline (*p* = 0.*06*) and at 1-year (*p = 0.09*) no statistically significant difference in cognitive impairment was observed between both groups. At year 2, a significant proportion of individuals in the non-CA group are cognitively impaired compared to the CA group (*p = 0.0002)*.

**Figure 2:**
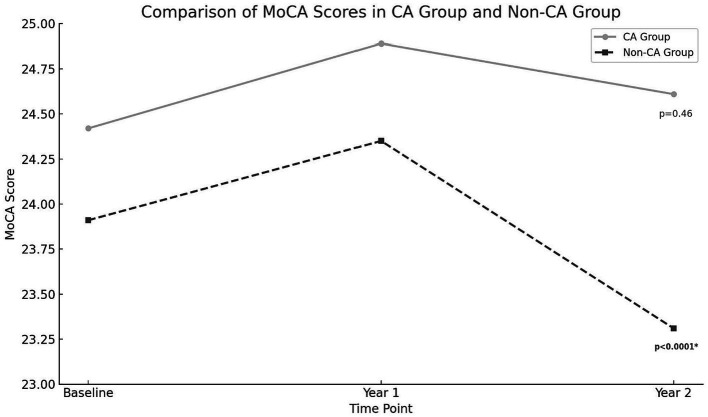
MoCA score at baseline, 1 and 2-year endpoints in both the CA and non-CA groups. A statistically significant drop in the MoCA score was observed at the 2-year end point compared to baseline in the non-CA group (*p < 0.0001*).

There were no significant differences in rates of cognitive impairment at baseline between SAGE-AF participants who had undergone a CA procedure before enrollment vs. those who did not (*32.1%* vs. *39.5%, p = 0.06*). However, participants in the CA group were significantly less likely to be cognitively impaired over the 2-year study follow-up, even after inclusion in a model adjusting for propensity to undergo CA (*aOR_1_ 0.70, 95% CI 0.50–0.97*). A second model (Model 2) attenuated the association between CA and cognitive impairment to non-significance by adjusting for AF treatment strategy (rate vs. rhythm control) and maintenance of NSR from baseline to 2-years in addition to propensity score *(aOR_2_ 0.75, 95% CI 0.52–1.08)* (Table 2). Figure 1 provides a graphics depiction of the MoCA score at baseline, 1- and 2-year follow-up in both the CA and non-CA groups. The total number of individuals with a MoCA ≤ 23 at baseline, 1-, and 2-year follow-up are included in Table 3 and Figure 2. At baseline 62 individuals (32.1%) in the CA had cognitive impairment compared to 274 (39.5%) in the non-CA group (*p = 0.06*). At the 2-year end point 58 individuals (30.1%) in the CA group and 311 (44.8%) in the non-CA group had a MoCA ≤ 23 (*p = 0.0002*). Further longitudinal analysis of mean raw MoCA scores for each group is given in Supplementary Table 1. NSR status based on CA at baseline and the 2-year endpoint is provided in Supplementary Table 2. Some individuals were found to be in NSR at “year 2” and not at baseline. Others were found to be in NSR at both baseline and year 2 and have been labeled within the table accordingly.

**Table 2:** Odds (95% Cis) of cognitive impairment (MoCA ≤ 23) over 2-years by prior catheter ablation for AF status at study enrollment.

AF ablation	Odds ratio (95% CI)		
	Unadjusted	Model 1	Model 2
Yes (*n* = 193)	**0.61** (**0.44, 0.84**)	**0.70** (**0.50, 0.97**)	0.75 (0.52, 1.08)
No (*n* = 694)	*Reference*		

Model 1 adjusts for propensity score [sex, age, BMI, CHA_2_DS_2_-VASc score, AF type (paroxysmal, persistent, or permanent), education level, smoking status, history of hypertension, history of heart failure, history of diabetes, history of stroke, history of renal disease, history of sleep apnea, use of rhythm control drugs, use of rate control drugs, and use of oral anticoagulants]. Model 2 adjusts for Model 1 plus treatment group (rate vs. rhythm control) and maintenance of NSR. Statistically significant values bolded.

**Table 3:** Number of individuals with MoCA ≤ 23 at baseline, 1-, and 2-year follow-up.

Cognitive impairment (MoCA ≤ 23)	AF Ablation?		*P*-value
	Yes (*N* = 193)	No (*N* = 694)	
Baseline	62 (32.1)	274 (39.5)	0.06
Year 1	54 (28.0)	239 (34.4)	0.09
Year 2	58 (30.1)	311 (44.8)	**0.0002**

Statistically significant values bolded.

### 3.2 Major ischemic/hemorrhagic events in CA and medically managed AF patients

After adjusting for confounding variables, we noted no statistically significant association between prior CA and odds of major bleeding (*aOR 0.53, 95% CI 0.24–1.16*) or the pre-specified composite outcome of all-cause MACE (ischemic stroke, vascular death, MI, and major bleeding; *aOR 0.65, 95% CI 0.35–1.20*) over 2 years follow-up (Supplementary Table 3).

### 3.3 Subgroup analysis of warfarin vs. DOACs on cognitive impairment

We did not observe any significant difference in either the CA or the no CA groups between the use of warfarin vs. DOACs on cognitive function, even after adjusting for propensity to receive CA (Table 4). Notably, both the CA and non-CA groups had high rates of oral anticoagulant use, with nearly 90% of the CA group and 84% of the non-CA group being prescribed an oral anticoagulant throughout the study period.

**Table 4:** Odds of cognitive impairment over 2-years using Warfarin vs. all other AC.

Ablation group	Odds ratio (95% CI)		
	Unadjusted	Model 1	Model 2
CA (*n* = 193)	0.80 (0.43, 1.48)	0.78 (0.41, 1.46)	0.77 (0.41, 1.43)
No CA (*n* = 694)	1.07 (0.76, 1.50)	1.02 (0.72, 1.43)	0.97 (0.69, 1.36)

Model 1 adjusts for propensity score (sex, age, BMI, CHA_2_DS_2_-VASc score, AF type (paroxysmal, persistent, or permanent), education level, smoking status, history of hypertension, history of heart failure, history of diabetes, history of stroke, history of renal disease, history of sleep apnea, use of rhythm control drugs, use of rate control drugs, and use of oral anticoagulants). Model 2 adjusts for Model 1 plus treatment group (rate vs. rhythm control) and maintenance of NSR.

## 4 Discussion

### 4.1 Atrial fibrillation and cognitive decline

In this retrospective analysis of an observational study that performed a longitudinal assessment of cognitive function, we found that participants who had undergone CA for AF at baseline were less likely to develop cognitive impairment over a 2-year follow-up compared to those who underwent medical rhythm or rate control, even after adjusting for propensity to undergo *CA.* These results are consistent with prior studies associating the persistence of AF with cognitive impairment, including an analysis that examined data from both the ONTARGET and TRANSCEND trials in 2012. In this study, the authors showed that AF was associated with an increased risk of cognitive decline (*HR 1.14, CI 1.03–1.26*), and incident dementia (*HR 1.30, CI 1.14–1.49*) (18). Our study is one of the few to demonstrate a link between CA and cognitive function among older AF patients using serial objective measures of cognitive health. The EAST-AFNET 4 trial observed no significant difference in MoCA scores at the 2-year follow-up. This contrasts with our findings, where participants who underwent catheter ablation were less likely to develop cognitive impairment.

Several factors may contribute to these differing outcomes. Firstly, the EAST-AFNET 4 trial employed a higher MoCA cut-off of 26 to define mild cognitive impairment, which differs from our cut-off value of 23. This discrepancy in cut-off points could potentially lead to different interpretations of cognitive impairment in the respective cohorts. Moreover, the EAST-AFNET 4 trial’s methodology and patient population may have varied from our study, potentially influencing the outcomes. Differences in the intensity of rhythm control strategies, baseline characteristics of participants, and the extent of continuous ECG monitoring could all contribute to the observed disparities. Furthermore, our study focuses on a specific cohort with certain demographic and clinical characteristics, which might not be entirely representative of the broader AF population. Thus, while our findings suggest a beneficial cognitive outcome associated with catheter ablation, they should be contextualized within the limitations of our study design and population, and the contrasting results of trials such as EAST-AFNET 4 (19).

AF is hypothesized to cause cognitive impairment primarily through cerebrovascular accident and cerebral microbleeds among other causes. Possible mechanisms include decreased cerebral blood flow, impaired autoregulation, endothelial dysfunction, and chronic inflammation (20). Disruptions in the former occur in AF and have been linked with a decline in cognitive function in patients with AF with or without a history of stroke (21, 22). In 37,025 prospectively analyzed patients without AF for 5 years, 10,161 (27%) developed AF and 1,535 (4.1%) developed dementia. AF has been independently associated with developing dementia with an increased risk of mortality (23–26).

It has been hypothesized that effective oral anticoagulation will therefore improve cognitive outcomes (27). Rhythm control through pharmacologic intervention or ablation has also been theorized to have benefits in cognition in AF through the prevention of rapid ventricular response (28). As mentioned, the non-CA group was more cognitively impaired at the 2-year endpoint compared to the CA group. These results are consistent with the AFFIRM study (29). Furthermore, there was no significant increase in both obvious ischemic/hemorrhagic events in patients who underwent *CA.* The major hypothesis for the observed cognitive benefit is due to a favorable hemodynamic profile in the cerebral microvasculature. Prior studies from the SAGE-AF cohort demonstrated a 9% comorbidity between frailty and cognitive impairment (30). We did observe a non-significant decrease in cognitive impairment in the CA group when controlling for NSR in addition to propensity matching (Table 2).

One study of cognitive outcomes utilizing MoCA score in AF patients who underwent ablation compared to a propensity-matched group who did not undergo ablation found that CA improved performance at 1 year. Similarly, in our cohort, a slightly higher MoCA score at the 2-year endpoint in the CA group (*24.42 ± 3.67* vs. *24.61 ± 3.71*). Another study utilized near-infrared spectroscopy to identify regional cerebral blood flow and cerebral activity in patients with persistent AF compared to post-ablation AF patients who maintained sinus rhythm found CA improved frontal and temporal brain activities in some patients and was associated with an improvement in depression and cognitive function (31, 32). Our results, in conjunction with previous studies, can help guide future research in identifying definitive biological mechanisms for the neurocognitive benefits of ablation. AF is associated with chronic inflammation, which may contribute to cognitive decline. Effective management of AF through CA might reduce this inflammation. By addressing the underlying arrhythmic issues in the heart, CA might improve overall cardiac function, leading to better systemic and cerebral circulation, thus potentially benefiting cognitive health (33).

In model 2, where we adjusted for AF treatment strategy (rate vs. rhythm control) and maintenance of normal sinus rhythm (NSR), the association between CA and reduced cognitive impairment was attenuated to non-significance. This finding could be interpreted to mean that the maintenance of NSR, rather than the method of achieving it (CA or medication), is a more crucial factor in preserving cognitive function.

The importance of maintaining NSR might be attributed to several mechanisms. Consistent NSR could result in improved cerebral hemodynamic profile and reduced incidence of micro-embolic events, both of which are implicated in cognitive decline. Additionally, NSR may be associated with lower levels of systemic inflammation, which has been linked to various neurocognitive disorders. These results suggest that future research and clinical strategies should perhaps focus more on the effective maintenance of NSR as a primary goal for preserving cognitive function in AF patients, rather than solely on the method of rhythm control. This would underscore the importance of comprehensive management strategies that not only address cardiac arrhythmia but also optimize overall cerebrovascular health.

### 4.2 Warfarin and OACs on cognitive function

The subgroup analysis revealed no difference in cognitive outcomes between those on warfarin and all other OACs. This result was observed in both AF patients who underwent CA and those who were only medically managed. Previous studies have shown benefits in the utilization of DOACs over warfarin, thought to be due to warfarin’s propensity to cause cerebral microbleeds (CMBs) and silent infarction. Studies comparing warfarin and DOACs reported that patients on DOACs for AF had a lower incidence rate of dementia when compared to warfarin. In our study, both groups were treated highly with anticoagulation due to their advanced age putting them at higher CHA_2_DS_2_-VASc scores. There was a higher proportion of patients receiving warfarin in the no-CA group (*63.1%* vs.*39.9%*). This could be interpreted to drive the elevated bleeding rates (*7.5%* vs.*4.2%*) and MACE (*10.4%* vs.*6.2%*) observed in the no-CA vs. CA groups, which was underpowered for significance. Our subgroup analysis argues against warfarin as a cause of elevated MACE, CMBs, or silent infarction as there was no difference in cognitive outcomes. Further prospective head-to-head comparative studies between specific anticoagulants are needed.

### 4.3 Strengths and limitations

The main strengths of our research are that the data were collected prospectively over 2 years. Furthermore, a validated standardized instrument (MoCA score) was used to examine cognitive function, enhancing reproducibility and sensitivity to subtle changes in cognitive function. However, our study also has several limitations. Our cohort included a smaller proportion of patients who underwent CA compared to those who were medically managed. Furthermore, we did not have a high number of participants who underwent medical rhythm control. We did not distinguish between the specific types of CA procedures or the number of recurrent CA procedures. Participants in our study were primarily non-Hispanic white and well-educated, which may impair the generalizability of our findings to other racial or ethnic groups or less well-educated AF patients. We were unable to compare interactions between CA, warfarin, and cognitive health in comparison to each DOAC as there were insufficient numbers of DOAC-treated patients in each treatment group. Observational studies detect associations and cannot establish causal relations between CA and cognitive health. However, we supported our observations with biologically plausible mechanisms and conducted rigorous propensity score adjustment. Other factors that should be considered when controlling confounders in future studies include occupational status in addition to education. It is pertinent to acknowledge that the time interval between undergoing catheter ablation (CA) and enrollment in our cohort was not analyzed in this study. This variable time interval could potentially impact the cognitive trajectories of participants, as the immediate and long-term effects of CA on cognitive function might differ. In future studies, a more detailed analysis of this time interval, along with its potential implications on cognitive function, should be considered to provide a more nuanced understanding of the relationship between CA and cognitive outcomes. Given continued concerns linking AF and vascular dementia, as well as other neurodegenerative diseases such as Alzheimer’s disease phenotyping patients is needed in future studies. This is ideally conducted using imaging biomarkers and machine learning models in conjunction with a battery of neuropsychological testing. This approach would enable researchers to differentiate between cognitive impairments of likely vascular origin and those without vascular changes. Such data would be invaluable in conducting post-hoc analyses to determine if the etiology of cognitive impairment modifies the relationship between AF and cognitive outcomes.

While our study provides valuable insights into the cognitive outcomes associated with catheter ablation in atrial fibrillation (AF) patients, we acknowledge a limitation in our methodology pertaining to the assessment of AF burden. The ECG monitoring strategy employed in this study involved routine ECG assessments at baseline and at 2-year follow-up, which may not fully capture the intermittent nature of paroxysmal AF. Consequently, our approach may have limited our ability to accurately quantify the AF burden and its potential relationship with cognitive function. Future studies would benefit from the utilization of continuous or long-term rhythm monitoring technologies, such as Holter monitors or implantable loop recorders, to provide a more comprehensive understanding of the AF burden and its impact on cognitive outcomes in this patient population.

## 5 Conclusion

Catheter ablation is widely performed to improve symptoms and reduce AF burden. Our findings suggest that adults with AF who undergo catheter ablation are also less likely to become cognitively impaired than those who receive medical treatment alone over a 2-year follow-up period. This is not explained by differences in rates or types of oral anticoagulants used between groups or differences in the characteristics of participants at baseline. Further contemporary studies including randomized controlled trials are needed to validate our findings and examine whether ablation technique (e.g., radiofrequency, cryothermal, pulse field), duration of the procedure or post-procedure anticoagulation use, or cerebral hemodynamics further influence cognition after CA among patients with AF.

## Data availability statement

The original contributions presented in the study are included in the article/Supplementary material, further inquiries can be directed to the corresponding author.

## Ethics statement

The studies involving humans were approved by University of Massachusetts Chan Medical School. The studies were conducted in accordance with the local legislation and institutional requirements. The participants provided their written informed consent to participate in this study.

## Author contributions

BS: Conceptualization, Data curation, Formal Analysis, Funding acquisition, Investigation, Methodology, Project administration, Resources, Software, Supervision, Validation, Visualization, Writing – original draft, Writing – review & editing. AH: Data curation, Formal Analysis, Methodology, Visualization, Writing – original draft, Writing – review & editing. PC: Conceptualization, Writing – original draft, Writing – review & editing. RA: Conceptualization, Investigation, Writing – original draft, Writing – review & editing. DL: Data curation, Formal Analysis, Investigation, Methodology, Software, Supervision, Visualization, Writing – review & editing. EO: Data curation, Methodology, Software, Writing – original draft. JM: Writing – review & editing. JS: Funding acquisition, Project administration, Writing – review & editing. DM: Conceptualization, Formal Analysis, Funding acquisition, Investigation, Project administration, Resources, Software, Supervision, Validation, Writing – original draft, Writing – review & editing. MM: Conceptualization, Formal Analysis, Funding acquisition, Investigation, Project administration, Resources, Supervision, Validation, Writing – original draft, Writing – review & editing.
